# Roll-designed 3D nanofibrous scaffold suitable for the regeneration of load bearing bone defects

**DOI:** 10.1007/s40204-016-0058-2

**Published:** 2016-11-18

**Authors:** Fatemeh Hejazi, Hamid Mirzadeh

**Affiliations:** Department of Polymer Engineering and Color Technology, Amirkabir University of Technology (Tehran Polytechnic), 424 Hafez Avenue, 1591634311 Tehran, Iran

**Keywords:** 3D scaffold, Coral microparticles, Cellular interactions, Mechanical properties

## Abstract

In this work, an innovative and easy method for the fabrication of 3D scaffold from 2D electrospun structures is introduced. For this aim, coral microparticles were fixed inside the nanofibrous PCL/Gelatin mat and the obtained structure was post assembled into a cylindrical design. Scaffold fabrication procedure is described in detail and morphological properties, physical and mechanical characteristics and in vitro assessments of the prepared scaffold are reported. Presences of coral microparticles in the structure led to the formation of empty spaces (3D pores) between nanofibrous layers which in turn prevent the compact accumulation of nanofibers. Post-assembly of the obtained nanofibrous coral-loaded structures makes it possible to prepare a scaffold with any desired dimension (diameter and height). Existence of coral particles within the nanofibrous mats resulted in distant placement of layers toward each other in the assembling step, which in turn create vacancy in the structure for cellular migration and fluid and nutrients exchange of the scaffold with the surrounding environment. Cell morphology within the scaffolds is investigated and cytotoxicity and cytocompatibility of the structure is evaluated using Alamar blue assay. Enhancement in mineralization of the seeded cells within the prepared coral-loaded scaffolds is demonstrated by the use of SEM-EDX. Performed compression mechanical test revealed excellent modulus and stiffness values for the cylindrical samples which are comparable to those of natural bone tissue.

## Introduction

To the regeneration of a damaged tissue, in particular bone tissue, the key factor is to develop a three dimensional scaffold which mimics the microstructure, chemical composition and mechanical and functional properties of native tissue. In this work, we aimed to develop a scaffold for the regeneration of load bearing bone tissue.

Cortical bone, synonymous with compact bone, is one of the two types of osseous tissue that form bones. Cortical bone facilitates bone’s main functions: to support the whole body, protect organs, provide levers for movement, and store and release chemical elements, mainly calcium. As its name implies, cortical bone forms the cortex, or outer shell, of most bones. Compact bone is much denser than cancellous bone, which is the other type of osseous tissue. Furthermore, it is harder, stronger and stiffer than cancellous bone. Cortical bone contributes about 80% of the weight of a human skeleton. The primary anatomical and functional unit of cortical bone is the osteon (Netter 1987). The osteon consists of a central canal called the osteonic (haversian) canal, which is surrounded by concentric rings (lamellae) of matrix which are composed of collagen nanofibers. Between the rings of matrix, the bone cells (osteocytes) are located in spaces called lacunae. Small channels (canaliculi) radiate from the lacunae to the osteonic (haversian) canal to provide passageways through the hard matrix. In compact bone, the haversian systems are packed tightly together to form what appears to be a solid mass (Cancer Registration and Surveillance Modules 2000; Martini et al. 2011; Ch 2012).

In tissue engineering, full differentiation and normal biological activity of the cells requires the possibility of their growth in a three dimensional manner (Kazemnejad 2009; Li et al. 2003). As such, providing 3D scaffold with efficient porosity and pore size for freely and optimal cell migration is essential (Thorvaldsson et al. 2008).

Electrospinning is one of the beneficial techniques for the fabrication of assemblies with nanofibrous structure of native ECM. Flexibility of process, variety of the possible materials to be used, control over the morphology of the obtained nanofibers and probability of additive incorporation within the nanofibrous structure are among the advantageous of this method (Chomachayi et al. 2016).

However, small diameter of electrospun nanofibers results in low porosity and small sized pores of the obtained structure. In such condition, no cellular migration can occur into the inner layers (Khorshidi et al. 2015), which leads to 2D flattening of the growing cells on top of the exterior surface.

To overcome these limitation of electrospun structures many studies have been done such as; using the mixed structure of nano and microfibers (Thorvaldsson et al. 2008; Khorshidi et al. 2015; Pham et al. 2006), applying sacrificial agent between nanofibers (Kidoaki et al. 2005; Nam et al. 2007),stacking electrospun layers and membrane (Beachley et al. 2014; Madurantakam et al. 2013; Tong and Wang 2013), assembly of spinned nanofibers (Koh et al. 2010; Kim and Lee 2011), mechanical expansion of the electrospun mats (Shim et al. 2010),rolling or folding the fibers (Ru et al. 2013; Leung et al. 2012), changing the solution properties (Hejazi and Mirzadeh 2016), and using liquid flow in the spinning fiber path (Nguyen et al. 2012). Although these methods were more or less successful in the modification of electrospinning process, they raise other drawbacks for the obtained structure such as decrease in mechanical properties.

In this work, we introduced an easy method for the fabrication of 3D scaffold from electrospun structure which possesses modified porosity and enhanced pore size in addition to proper mechanical properties for load bearing applications. For this aim, coral microparticles were fixed within nanofibrous structure and the fabricated mat was rolled up into the cylindrical shape.

Natural coral is comprised of calcium carbonate (more than 98%) with highly porous structure possessing numerous interconnected channels (http://www.drugs.com/npp/coral.html). Due to its similarity to bone tissue, coral is compatible and has a good degradation rate that could well match the new bone formation rate in the application of bone tissue engineering (Xiao et al. 2010). Natural coral has good mechanical properties which are comparable to that of native bone tissue (John and Chamberlain 1978).

This work is the first report of using a mixed structure of polymer and natural coral. In fact, we have established this innovative method to make use of biopolymers advantageous in addition to natural coral benefits. Nanofibrous micro structure and advanced cellular interactions of polymer phase together with mechanical and osteconductive properties of coral, could help to achieve a proper scaffold for the regeneration of load bearing bone defects.

## Experimental

### Materials

Polycaprolactone (PCL), MW = 80,000 Da and gelatin from porcine skin, Type A, lyophilized powder, γ-irradiated, BioXtra, suitable for cell culture, were purchased from Sigma Aldrich. Acetic acid and ethyl acetate were purchased from Merck (Germany). Coral was obtained from Persian Gulf coral reefs.

### Coral sizing and disinfection

Coral pieces were grinded by the use of mechanical mill and the obtained particles were classified into different sizes by the use of sequential sieves.

After being sorted, the coral microparticles were disinfected by immersing in sodium hypochlorite solution (Merck, Germany) for 30 h followed by washing with distilled water several times and vacuum drying. It is worth to mention that the disinfection process of the coral was carried out after they were grinded into micro sized particles, to enhance the exposure of their surface with disinfecting solution. After being disinfected, coral particles were sterilized by being exposure to gamma radiation (25 KG) for 2 h.

### Scaffold fabrication method

16 wt% polymer solution for electrospinning was prepared by dissolving PCL and gelatin with a weight ratio of 70:30 in a solvent mixture containing acetic acid, ethyl acetate, and water in 3:2:1 ratio. 4.8% concentrations of gelatin was taken in a glass container containing solvent and dissolved at 50 °C under constant stirring for 4 h. After the gelatin was dissolved, PCL (11.2 wt%) was added to this solution and was stirred overnight at room temperature. (Binulala et al. 2014).

The prepared polymer solutions were taken in a 5 ml syringe and loaded in the electrospinning setup. Electrospinning was performed with an applied voltage of 10 kV, flow rate of 1 ml/h and the needle tip and collector distance (air gap) of 10 cm, to a grounded metallic rotating mandrel for 1 h.

**Fig. 1:**
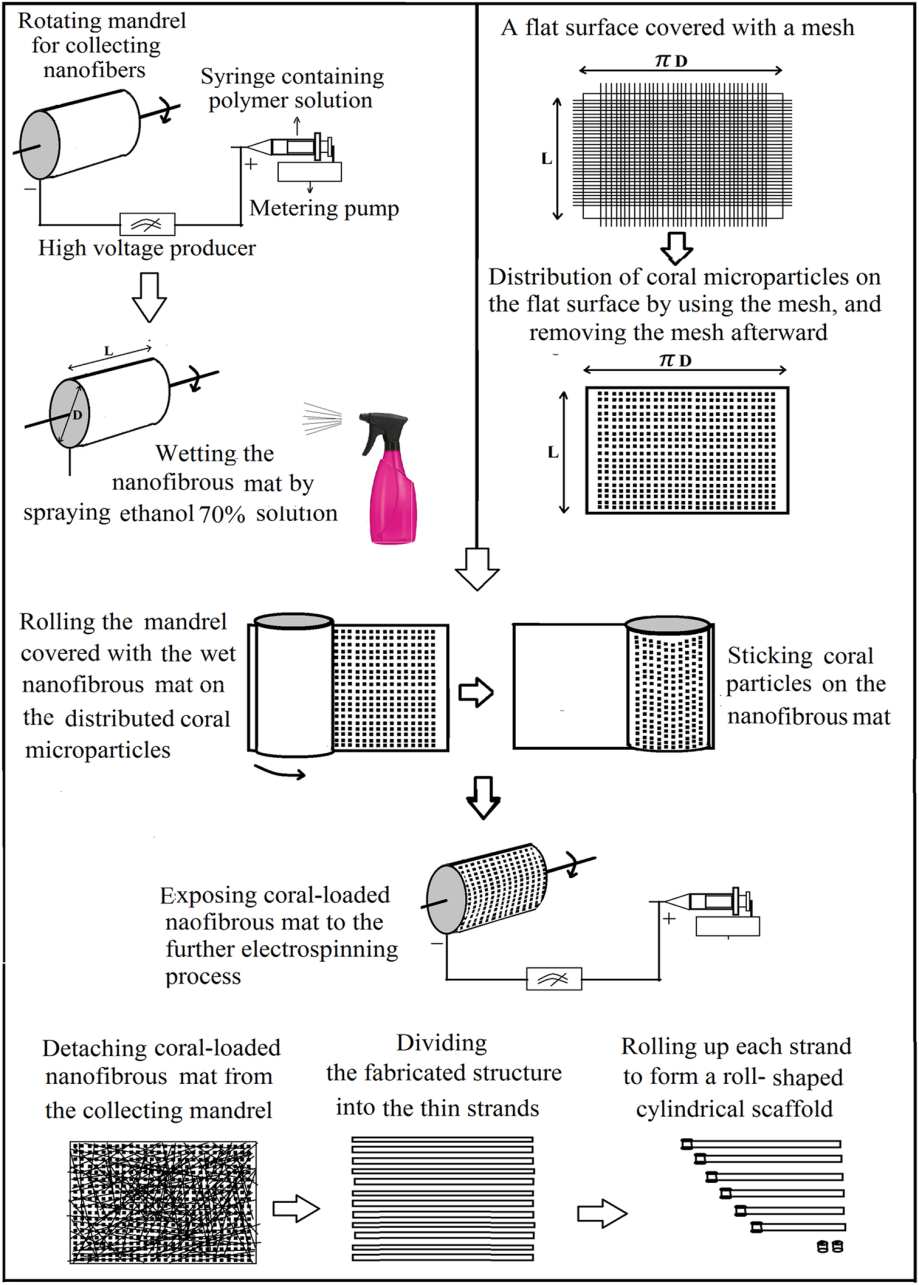
Schem of the step by step procedure used for the fabrication of coral-loaded roll scaffolds

For the preparation of coral containing structures, ultimate weight ratio of polymer to coral in the structure was considered as 1:1 or 1:2. At desired time intervals (one time after 30 min of electrospinning for obtaining 1:1 ratio, and two times every 20 min for 1:2 ratio) a defined amount of coral microparticles was loaded on the collected nanofibrous mat (Fig. 1).

To obtain uniform distribution of coral microparticles, first the particles were dispersed on a flat surface according to a mesh pattern (Fig. 1). For this aim, a flat rectangular plate which its width and length were equal to the length (*L*) and circumference (*πD*) of the rotational mandrel, respectively, was used as the substrate. For primary distribution of coral microparticles on this plate, a mesh (No 140, 70 and 50, for distribution of microparticles with the size of 100, 200 and 300 μm, respectively) was put on it. A defined amount of coral (equal to the overall weight of the nanofibrous layer that would form after 1 h of electrospinning) was dispersed on the plate which was covered with mesh. After picking up the mesh from the plate, a uniform distribution of particles was obtained according to the pattern of the mesh pores.

At each considered time interval, the formed nanofibrous layer was wetted by spraying ethanol (70%) solution. The collector mandrel which was covered by the wet nanofibrous mat was removed from the electrospinning chamber and put on the explained plate and rolled on it from one side to the other. By this method, coral microparticles were attached to the wet surface of nanofibrous mat with a uniform pattern. The mandrel which was covered with coral-loaded nanofibrous mat was returned to the electrospinning chamber and exposed to the further spinning procedure.

To prepare the 3D scaffolds, the fabricated electrospun mats (with or without coral microparticles) were cut into strands with desired width and length, then these strands were rolled up into cylindrical shape. To prevent the prepared structure from unrolling, the ultimate (outer) layer was attached to the former layer by a mild and quick exposure of a small spot in the tail-end of outer layer of the rolled structure to a hot surface. Seven types of roll scaffolds were prepared by varying the polymer to coral ratio and coral particle size (Table 1).

**Table 1:** Prepared samples codes and composition

Sample code	Polymer to coral ratio	Coral microparticle size (μm)
CF-Roll	1:0	No loaded coral
Roll A	1:1	100
Roll B	1:1	200
Roll C	1:1	300
Roll D	1:2	100
Roll E	1:2	200
Roll E	1:2	300

### Scaffold morphology

The morphological evaluation of the scaffolds was performed with SEM (AIS 2100, Seron Technology, Korea) after sputter coating (BAL TECH) with gold.

### Porosity measurement

To measure the porosity of the prepared scaffolds, two methods were considered.

#### Gravimetry method

In the first step, porosity of the prepared samples was obtained from gravimetry method by the following equation (Szentivanyi et al. 2011):

1 $$ {\text{Porosity }}\left( \% \right) \, = { 1} - \frac{{\rho_{\text{s}} }}{{\rho_{\text{mix}} }} \times 100 = { 1} - \frac{{{\raise0.7ex\hbox{${W_{\text{s}} }$} \!\mathord{\left/ {\vphantom {{W_{\text{s}} } {V_{\text{s}} }}}\right.\kern-0pt} \!\lower0.7ex\hbox{${V_{\text{s}} }$}}}}{{\rho_{\text{mix}} }} \times 100 $$

where $W_{\text{S}}$ represents the scaffold dry weight, $V_{\text{s}}$ is the volume of the scaffold and $\rho_{\text{mix}}$ stands for the density of the used materials which determines with the following equation (Feinstein 1972):

2 $$ \rho_{\text{mix}} = \frac{{M_{\text{T}} }}{{\left( { V_{\text{A}} + V_{\text{B}} } \right)}} $$

where $M_{\text{A}}$ and $M_{\text{B}}$ are mass of A and B in the mixture, $V_{\text{A}}$ = $\frac{{M_{\text{A}} }}{{\rho_{\text{A}} }}$, and $V_{\text{B}}$ = $\frac{{M_{\text{B}} }}{{\rho_{\text{B}} }}$.

#### Mercury intrusion method

Mercury intrusion porosimetry was the second method which was considered to determine scaffold porosity and pore size.

In this method, mercury, which was a nonwetting liquid, entered through the dry scaffold under pressurized conditions. Samples placed in penetrometer and subjected to high vacuum. By achieving the minimum required pressure inside the penetrometer, mercury flowed and surrounded the sample. Subtracting the initial volume of the mercury which surrounds the sample from the volume of the penetrometer gave the sample volume ($V_{\text{scaffold}}$). By increasing the applied pressure, the mercury entered increasingly smaller pores. Total volume of mercury forced into the sample was defined as $V_{\text{intrusion}}$. As such, the open porosity of the scaffold (*π*), which is accessible for the intrusion of mercury, was calculated from the following equation (Karageorgiou and Kaplan 2005; Loh and Choong 2013):

3 $$ \pi = \, \left[ {\frac{{V_{\text{intrusion}} }}{{V_{\text{scaffold}} }}} \right] \times 100 $$

And the closed porosity (*ω*), pores with no intery in which the mercury can not diffuse through them, was calculated by the use of total porosity (*P*) which was determined by gravimetry method.

4 $$ \omega \, = \, P \, {-} \, \pi $$

The volume of the diffused mercury at each pressure interval was used for the estimation of pore size. The pore size was estimated using the Washburn equation:

5 $$ r = \frac{{2s { \cos }\left( \theta \right)}}{p} $$

where *r* is the pore radius, *s* is the surface tension of mercury, *θ* is the contact angle of the mercury, and *p* is each pressure interval. Radius values were calculated automatically and average value was obtained after all points were recorded.

For all kind of samples, 3 individual replicants were used and the results reported as the average value.

### Water uptake test

The water uptake capacity of the prepared scaffolds was determined by soaking them (*n* = 3) in phosphate buffered saline (pH 7.4) at 37 °C for for 0.5, 1, 6, 24, 96 and 240 h. The water-uptake ratio is defined as the ratio of the weight increase to the initial weight ($w_{\text{d}}$) (Pezeshki-Modaress et al. 2014).

6 $$ W = \, [(w_{\text{w}} - w_{\text{d}} )/w_{\text{d}} ] \times 100 $$

where $w_{\text{w}}$ represents the weight of the scaffolds after immersion in the PBS solution at each time point and $w_{\text{d}}$ is the initial weight of the dry scaffolds. For all kind of samples, 3 individual replicants were considered and values are expressed as the mean ± standard deviation.

### Mechanical analysis

Uniaxial mechanical tests were performed with (SANTAM, STM-20) in z-directional (along with the cylinder axis) compression mode, at a crosshead rate of 0.5 mm/min, and 0.05 N preload. 3D samples (*n* = 3, *Ø* = 5 mm, *h* = 10 mm) were tested in dry condition and compressive load was applied up to 50% deformation. Compressive modulus and stiffness were drawn from the stress–strain curve elaboration. Data are reported as average ± standard deviation.

### MG63 cell preparation

In this study, MG-63 Human osteosarcoma cell line was used for in vitro assays. MG-63 cells were cultured in DMEM/F12 medium containing 10% FBS, 1% l-glutamine, and 1% penicillin/streptomycin in T-75 tissue culture flask, and the culture medium was refreshed every 3 days. Cells between three and four passages were used for the experiments. Prior to each cell seeding, dissociated cells (with 0.05% trypsin/EDTA) were centrifuged, and then resuspended in fresh medium.

### In vitro cytotoxisity evaluation

The possible release of low molecular weight cytotoxic substances from the samples and also coral microparticles were investigated by indirect cytotoxicity tests, using MG63 human osteosarcoma cells line. Cylindrical samples and coral microparticles (after being disinfected) were sterilized by exposure to Gama irradiant for 120 min. To obtain the extracts, three specimens from each considered sample were immersed in DMEM medium supplemented with 10% fetal bovine serum (FBS) and 1% penicillin/streptomycin. Non-interfered complete medium was considered to prepare the control. 24 h before adding the extract, MG63 cells were seeded (cell density = 2 × 10^4^ cells/well) in a 96-well tissue culture plate. After 1, 4 and 7 days of incubation, the extracts and non-interfered mediums were removed and replaced the medium of pre-seeded cells. Three replicates for each eluate and medium were considered. After 24 h, the culture medium of each well was replaced with 150 μl Alamar Blue™ solution (10% v/v in culture medium) and the plate incubated for 4 h. 100 μl from the solution of each well was transferred to a 96 well plate and the absorbance measured using Thermo Scientific Multiscan Spectrum plate reader (test wavelength: 540 nm; reference wavelength: 630 nm). Reported percentage is relative ratio of values related to the cells in contact with samples to the cells in contact with non-interfered medium.

### Cell seeding

MG-63 cells were resuspended in growth medium containing 10% FBS at the density of 7 × 10^5^ (cells/ml). To enhance cell attachment to the samples, 100 μl of cell suspension was put directly onto the top of each scaffold to obtain a total number of 7 × 10^4^ cells/scaffold and the samples were put in incubator. After 1.5 h each well was filled up with the medium. The growth medium was replaced regularly every 2 days.

### Cell morphological observation

At considered time points for morphological investigation (4 h, 1, 3, 5, and 7 days), cell cultured samples were detached, washed with PBS solution and fixed in 4% glutaraldehyde solution for 30 min. After cell fixation, samples were washed with PBS buffer again to remove residual glutaraldehyde followed by dehydration in sequentially increasing ethanol solutions (10 min for each concentration). Specimens were subsequently dried in vacuum overnight. Prepared samples were gold sputtered and observed under scanning electron microscope (AIS 2100, Seron Technology, Korea).

### Histological evaluation

After In Vitro cell culture, at desired time points, the scaffolds were fixed with a formalin10% solution and embedded in paraffin. Paraffin blocks were sectioned (5 μm thick), placed onto charged glass slides, deparaffinized and rehydrated. For histological analysis, sections were stained with hematoxylin and eosin (H&E) to visualize cell infiltration and distribution using optical microscope.

### Cell proliferation

To evaluate the MG-63 cells proliferation within the prepared scaffolds, direct cytocompatibility test was performed in considered time points. For this aim, cylindrical samples (*D* = 15 mm, *h* = 5 mm) were cell seeded (with the density of 7 × 10^4^ (cells/well)) in 24-multiwell tissue culture plate up to 21 days. At each time point, cells viability (which is a sign of cell number at each time interval) was determined by replacing culture medium with 2 ml AlamarBlue™ solution (10% v/v in culture medium). After 4 h, solution from each well was transferred to 3 wells of 96-multi well plate (100 μl in each well) and fluorescence value was measured at (test wavelength: 540 nm; reference wavelength: 630 nm). Average value related to each sample, was normalized by dividing to the average fluorescence value of the controls. Subsequently, scaffolds were washed with PBS, fresh culture medium was added and the plate was transferred to the incubator for the next time-point.

### MG-63 cell mineralization

Calcium formation on the MG-63 cell seeded scaffolds, was identified by SEM instrument (VEGA, TESCAN, Czech) equipped with energy dispersive X-ray (EDX) spectrometer.

### Statistical analysis

Statistical analysis was performed using One Way ANOVA analysis of variance (SPSS 16.0 software) followed by Tukey’s significant difference post hoc test. Experiments were performed in triplicate and data are expressed as mean ± standard deviation. A *p* value <0.05 was considered statistically significant.

## Results and discussion

### Morphological evaluation of the scaffold

**Fig. 2:**
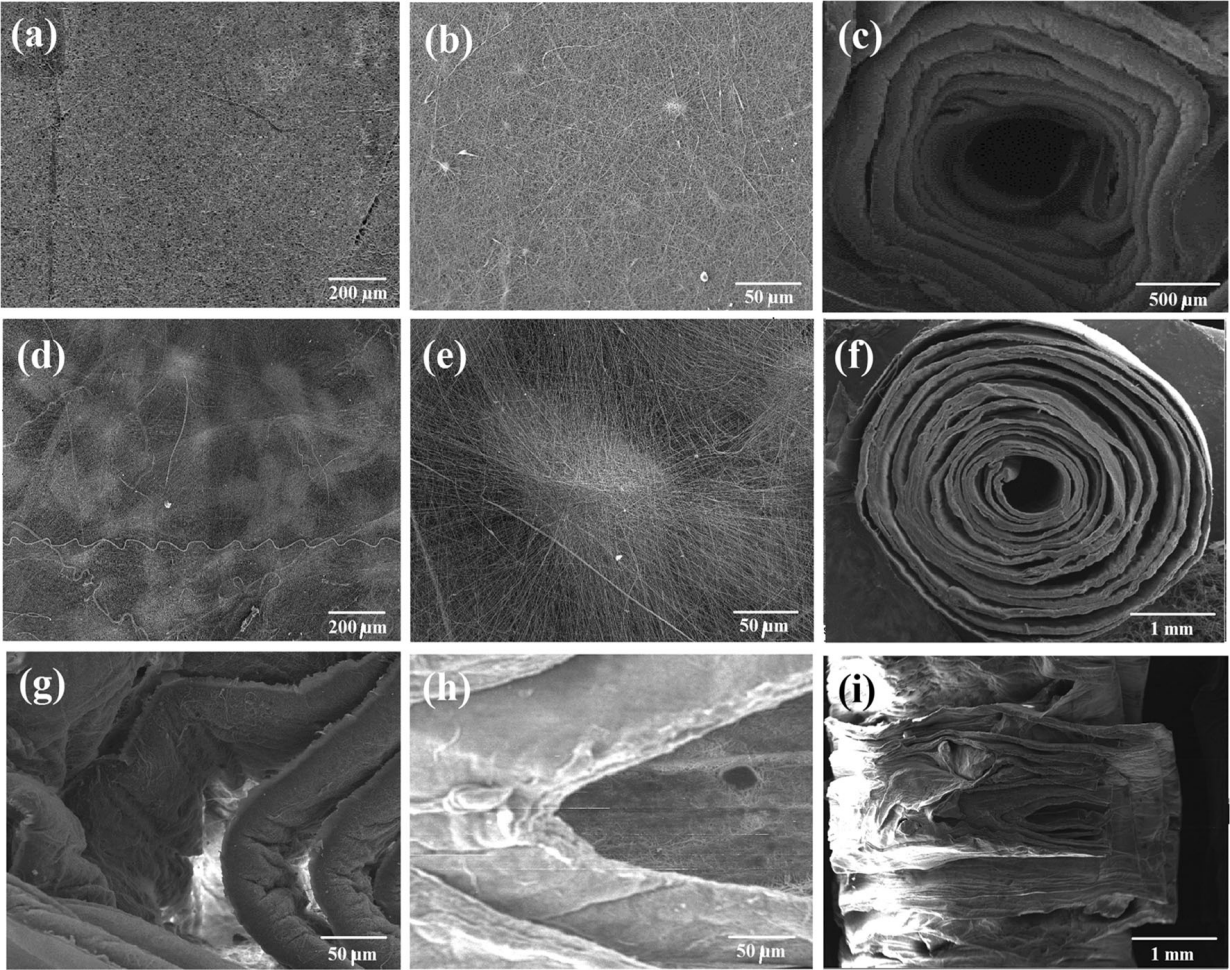
Scanning electron microscopic images of the prepared structures. **a**–**c** Conventional PCL/Gelatin (PG) nanofibrous mat and the related roll scaffold (CF-Roll). **d**–**f** Coral micro particle-loaded PG nanofibrous mat and the related roll scaffold. **g**–**i** Cross sectional view of the coral-loaded rolls

Figure 2 shows the SEM micrographs of the prepared structures. The compact accumulation of nanofibers in 2D electrospun mat is illustrated in upper lane, while existence of coral microparticles between the spun nanofibers formed a porous structure with non-compact nanofibrous layers (second lane). Top views of the rolled coral-free and coral-loaded nanofibrous layers are shown in Fig. 2c, f, respectively. Horizontal and vertical cross sections of the rolled scaffolds are shown in Fig. 2g, h in addition to focus on inner region of vertical cross section (i). These cross-sectional images illustrate the distant placement of nanofibrous layers as the result of the presence of coral microparticles.

### Porosity and pore size evaluations

Porosity of the prepared scaffolds was obtained by gravimetry and mercury intrusion methods and the calculated values were summarized in Table 2. According to the reported data, although CF-Roll (which contains no coral microparticles) possesses high total porosity value, the major porosity percentage relates to the closed pores. Existence of coral microparticles in the structure led to the enhancement of open porosity value by preventing the dense deposition of nanofibers toward each other. By increase in the coral ratio (without changing the particle size) total porosity value decreased as the result of increased amount of coral microparticles which possess higher density compare to polymer phase. In addition, open porosity value also decreased because of the occupation of free spaces by additional coral microparticles. Increase in coral particle size, led to the enhancement of both total and open porosities by enlarging the free spaces in the structure.

**Table 2:** Porosity values of the prepared structures obtained by porosimetry methods

Porosity sample	Total porosity (gravimetry method) (%)	Open porosity (mercury porosimetry) (%)	Close porosity (mercury porosimetry) (%)
CF-Roll	56.2 ± 5.2	35.1 ± 0.5	21.1
Roll A	39.1 ± 3.5	35.7 ± 0.4	3.4
Roll B	60.7 ± 4.6	57.8 ± 0.7	2.9
Roll C	69.4 ± 6.3	67.1 ± 0. 4	2.3
Roll D	28.7 ± 3.2	24.5 ± 0.3	4.2
Roll E	53.3 ± 5.7	49.6 ± 0.6	3.7
Roll F	61.5 ± 4.9	58.4 ± 0.4	3.1

Average pore size and pore size ranges of the prepared scaffolds are summarized in Table 3. According to the reported data, presence of coral microparticles in roll structures led to the formation of significantly bigger pores compare to the pores of coral-free rolls. Increase in the coral size is simultaneous with the increase in average pore size and wider pore range. The observed decrease in the pore size values when coral percentage was doubled could be explained due to denser population of microparticles which occupied the free spaces between nanofibrous layers.

**Table 3:** Mercury porosimetry results of pore characteristics of the prepared scaffolds

Sample	CF-Roll	Roll A	Roll B	Roll C	Roll D	Roll E	Roll F
Average pore size (μm)	8.32 ± 1.45	74.6 ± 4.6	159.48 ± 8.44	243.83 ± 10.6	51.70 ± 5.3	123.89 ± 7.73	217.47 ± 12.65
Pore size range (μm)	1–18	2–126	1–208	2–312	1–106	2–230	1–304

### Water uptake

**Fig. 3:**
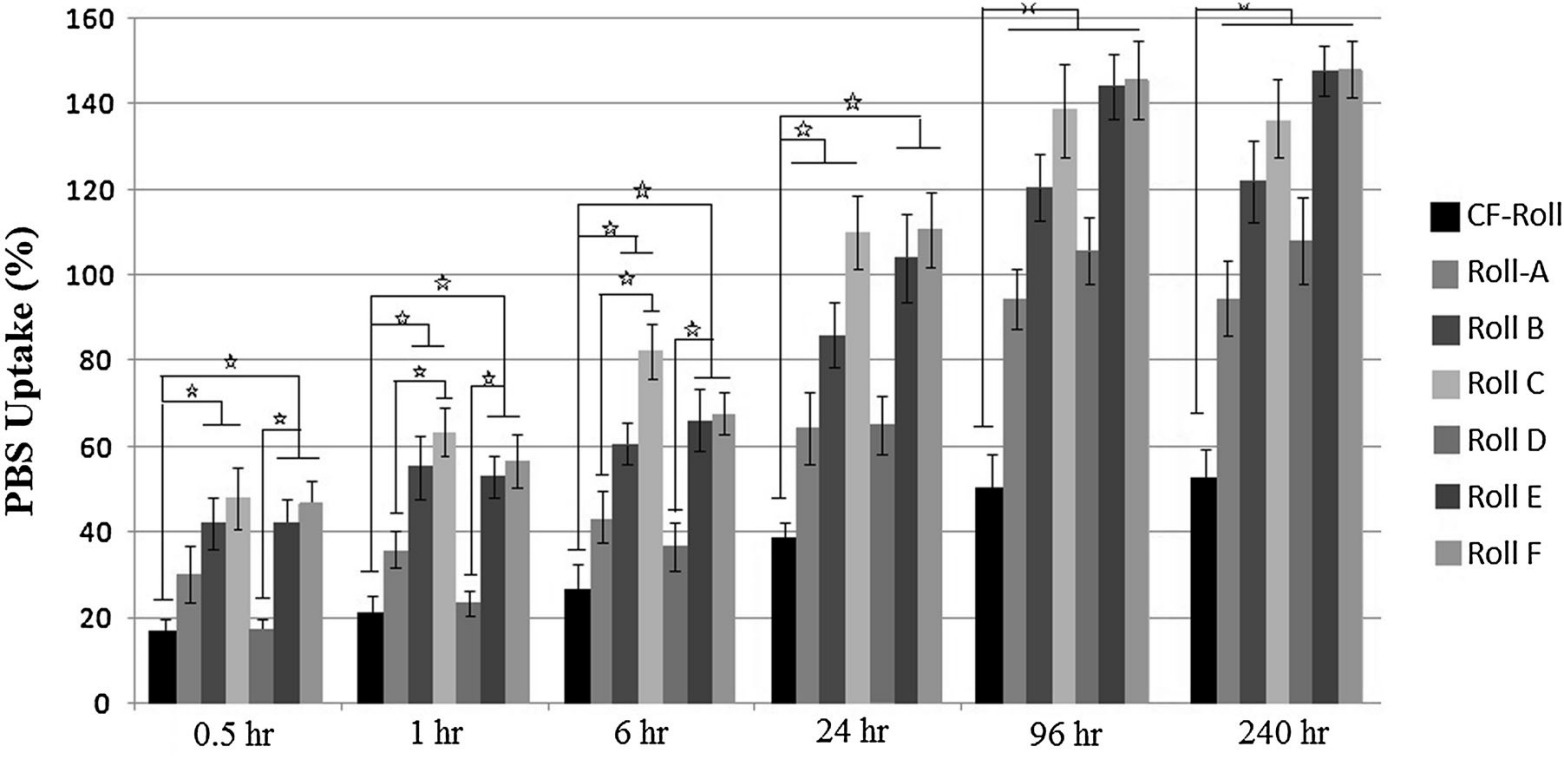
Water uptake behavior in PBS solution up to 240 h for different types of Roll scaffolds. Statistically significant difference is denoted as * (*p* < 0.05)

Figure 3 illustrates the water uptake trends of the prepared roll scaffolds over the considered time points. Comparing the PBS uptake values of coral-loaded specimens to that of the coral-free samples, Rolls containing coral microparticles exhibited significantly higher values (*p* < 0.05) than coral-free ones. In addition, remarkable increase in the water uptake values was detected between the successive time points for coral-loaded samples up to 240 h, whereas for the coral-free samples slight increase was observed.

These results can be correlated to the increased open porosity of coral containing structures compared to the compact coral-free rolls. PBS solution absorption occurred in two different ways, pores absorption and polymer absorption. Presence of more percentage of open porosity, allowed for increased amount of diffused solution into the inner regions of the scaffold. On the other hand, much more amount of diffused solution within the structure enhanced the exposure of inside polymer nanofibers with the fluid, which in turn increased the polymer water absorption. Improvement in the explained water absorption possibilities (pore absorption and polymer absorption) led to the increase in overall PBS uptake values of coral containing rolls.

Increase in coral microparticle amount in the structure, decreased the water uptake rate at early time points because of initial inhibition toward solution diffusion as the more microparticles act as barrier in the way of interring solution. While at further time points, when slow but continuous diffusion was occurred, because of more distant placement of nanofibers toward each other as the result of more in-between microparticles, exposure of nanofibers and PBS solution intensified and overall PBS uptake values increased because of more polymer adsorption possibility. Acoording to the reported data, for all of the prepared scaffolds no remarkable increase in PBS uptake was observed between 96 and 240 h which indicates equilibrium point for water uptake capasity.

Increase in coral particle size in addition to enhancing the porosity (which led to the more pore absorption) of the scaffolds, formed bigger pores in the structure that increased the PBS uptake rate from early time pints, which in turn led to the more and accelerated polymer absorption.

### Mechanical analysis

**Fig. 4:**
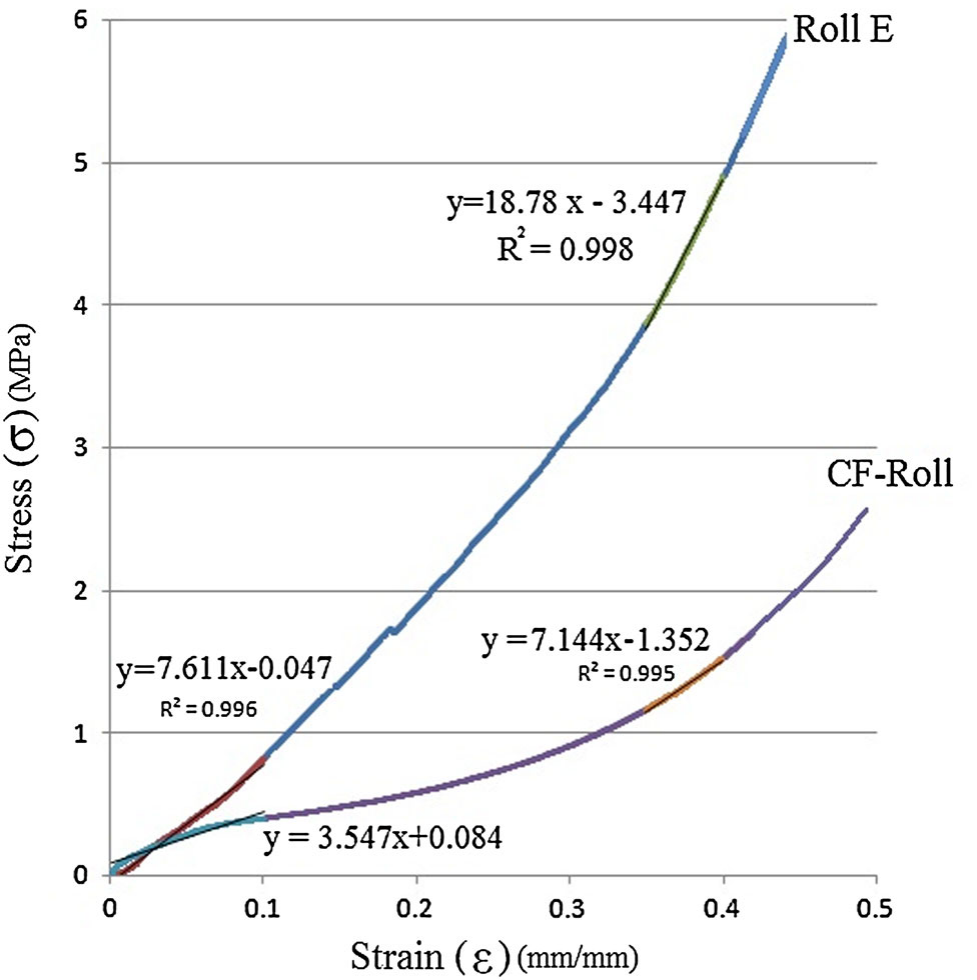
Representative compression stress–strain curves of Roll E specimens

To evaluate the mechanical properties of the prepared 3D nanofibrous scaffolds, compressive mechanical tests were performed along with the cylindrical axis of the samples. For each sample, the compressive load was applied up to 50% deformation and representative stress–strain curves were drawn. Figure 4 represents the σ/ε curves related to coral-free verses coral-loaded (1:1, 200 μm) roll. The specific structure of rolled nanofibrous mat led to obtaining high modulus and stiffness for CF-Roll scaffold compared to other reported nanofibrous structures (Shim et al. 2010; Nguyen et al. 2012; Jennes et al. 2012; Gu et al. 2013; Sawawi et al. 2013; Lee et al. 2011; Sun et al. 2012; Wulkersdorfer et al. 2010; Kim et al. 2014; Baker et al. 2012; Milleret et al. 2011). On the other hand, by the incorporation of stiff coral particles into the polymeric structure, the σ/ε curve moved up to higher stress values and the resultant compressive modulus and stiffness values increased significantly. Compressive mechanical properties of different types of roll samples are summarized in Table 4.

**Table 4:** Compressive mechanical parameters determined from stress–strain curves of the compression tests performed on the 3D cylindrical Roll scaffolds

Sample	Modulus (GPa)	Stiffness (GPa)	σ (50%) (Mpa)
CF-Roll	3.547 ± 0.564	7.144 ± 1.842	2.568 ± 0.715
Roll A	3.052 ± 0.976	10.867 ± 2.379	3.802 ± 0.830
Roll B	2.829 ± 0.713	11.136 ± 1.830	4.058 ± 0.964
Roll C	2.302 ± 0.609	12.110 ± 2.461	4.840 ± 0.776
Roll D	8.247 ± 1.476	17.562 ± 3.679	7.250 ± 1.593
Roll E	7.611 ± 1.057	18.78 ± 3.096	6.928 ± 1.830
Roll F	7.009 ± 0.942	20.62 ± 3.558	7.603 ± 1.761

According to the reported data, existence of coral microparticles within the polymer phase with the ratio of 1:1, led to the slight decrease in compression modulus in small strains. This behavior is mainly related to the collapse of the empty spaces which were formed as the result of microparticle existence within the scaffold.

In the higher deformations the structure was more compact, so it was the coral phase which resisted toward the applied force. As such the significant higher stiffness and σ_50%_ values (*p* < 0.05) were detected for coral containing structures compared to coral free ones.

By increasing the coral ratio (from 1 to 2), significant increase in modulus as well as stiffness and σ_50%_ was observed. This observation can be explained due to the increased percentage of stiff coral phase which resisted toward applied force from small deformations.

Increase in coral particle size has dual effect on the mechanical properties. Increase in the porosity value by bigger coral microparticles led to the decrease in mechanical properties, while the required extra force to break up the bigger particles, increase the mechanical properties of the samples. In lesser quantities of coral and smaller deformations, the first effect is more prominent, but with the further coral ratio and also in higher deformations, the latter effect would be prevailing.

### In vitro cytotoxicity evaluation

**Fig. 5:**
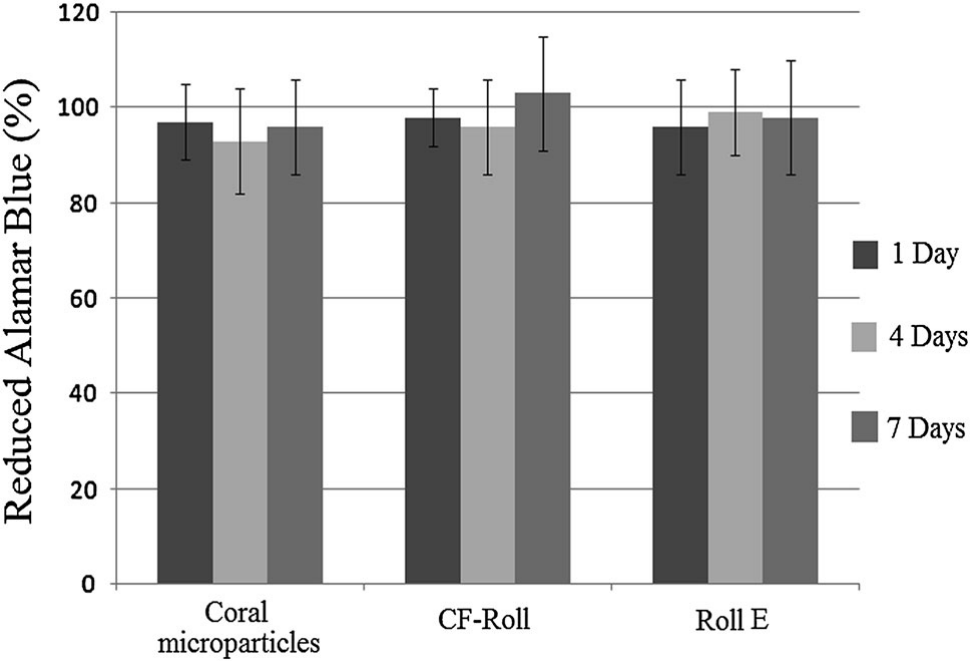
Values of Alamar Blue reduced by MG63 cells cultured in contact with 3D Roll sample eluates at 1 and 4 and 7 days. All the values are expressed as percentage versus the control

DMEM extracts were obtained by immersion of samples and also coral particles in complete culture medium for 1, 4 and 7 days. The values of Alamar Blue reduced by MG63 cells cultured in presence of the extracts were normalized to the Alamar Blue values related to the cells in fresh DMEM incubated for 1, 4 and 7 days. The obtained results (Fig. 5) indicated good cell viability for the cells which were cultured in contact with the extracts of the samples as well as coral microparticles. Considering the eluates of three time points, no significant difference (*p* > 0.05) was observed for the samples and also coral microparticles.

### Cell morphology

**Fig. 6:**
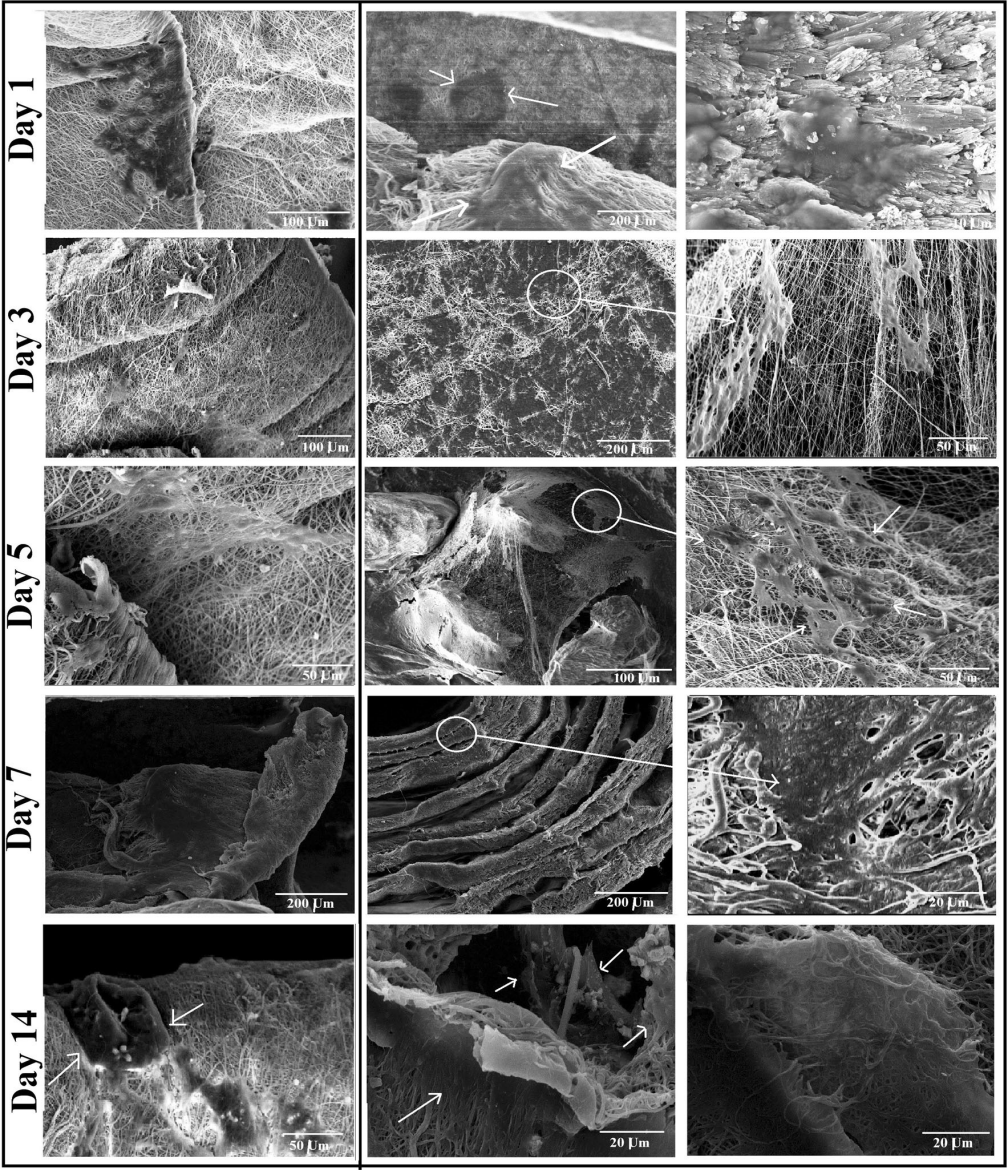
SEM images of MG-63 cells seeded scaffolds at different time points. *First column* represent the cell seeded CF-Rolls. *Second* and *third column* shows different internal regions of the cell seeded coral-loaded rolls

Figure 6 shows the SEM images of MG63 cells seeded on the CF-Roll (First column) and Coral-loaded Roll (second and third columns) scaffolds at different time points (SEM images are related to the midline horizontal and vertical cross sections of the scaffolds). As is illustrated in these images, seeded cells were detectable on the exterior surface of CF-Rolls with no considerable migration due to the compact arrangement of the nanofibrous layers. Instead, on the coral-loaded rolls, cells spreading and migration into the inner regions of the scaffold were remarkable, because of the enhanced pore size and open porosity between nanofibrous layers as the result of in-between coral microparticles. In this structure, cells can migrate between nanofibrous surfaces and in addition to being in contact with the nanofibrous substrate, can bridge the surrounding nanofibrous walls instead of just flattening on the surface.

### Cell proliferation

**Fig. 7:**
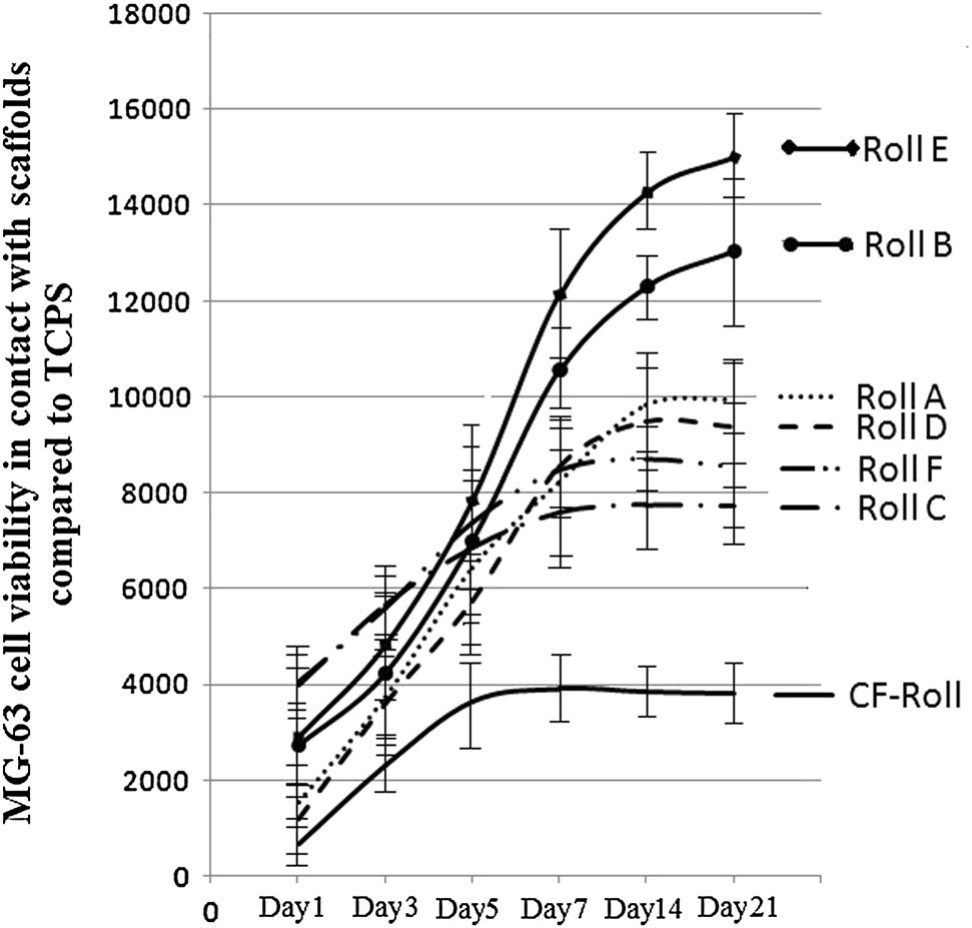
Viability trend of MG-63 cell at the considered time points in the case of being in contact with each scaffold type

MG63 cells were seeded onto the roll structures up to 21 days; Alamar Blue biochemical assay was performed to investigate cell viability on the samples at different time points and the related results are reports in Fig. 7. Compared to the coral-loaded scaffolds, coral-free roll shows lower cell viability and early stage stop in proliferation which indicates that the cells did not diffuse into the inner regions and reach to confluence on the exterior surface of the scaffold in a short period of time. At the existence of coral microparticles, by increase in open porosity of the scaffolds, the probability of cell diffusion enhanced, as such the proliferation rate and ultimate viable cells on the related rolls increased as a result of increased area for cell growth. Increase in coral particle size led to the higher proliferation values at early time points. This observation may be due to the fact that bigger particles provide bigger pores which ease the cellular diffusion into the structure. But at further time points, when the cells completely migrate throughout the scaffolds, this is the probability for the cells in bridging over the nanofibrous layers which effect the proliferation value. According to the results, by the use of microparticles with 200 μm average diameter (Roll B and Roll E), the best situation for cell migration and growth was provided and subsequently, proliferation value is significantly higher than when microparticles with 100 μm diameter was used (Roll A and Roll D). But when the particles with 300 μm diameter is used (Roll C and Roll F) a reverse phenomenon was observed. This observation could be explained due to the fact that the distance between fibrous layers got too big and it is not possible for the cells to bridge over, hence affecting cell growth negatively.

Increase in coral percentage has dual effect on the cell proliferation values of prepared scaffolds. By playing as barriers toward cellular migration, the additional coral particles would decrease the proliferation value. On the other hand, by playing as substrates for cell growth, further microparticles led to the enhancement of proliferation value. The first effect is dominant when smaller coral particles are applied (Roll D compared to Roll A), while by the use of bigger particles, the second effect is prevailing (Roll E compare to Roll B and Roll F compare to Roll C). According to the overall results, Roll E can be chosen as the optimum sample considering the appropriate porosity and pore size and the resultant enhanced tendency of the cells to growth within this structure.

### H & E staining

**Fig. 8:**
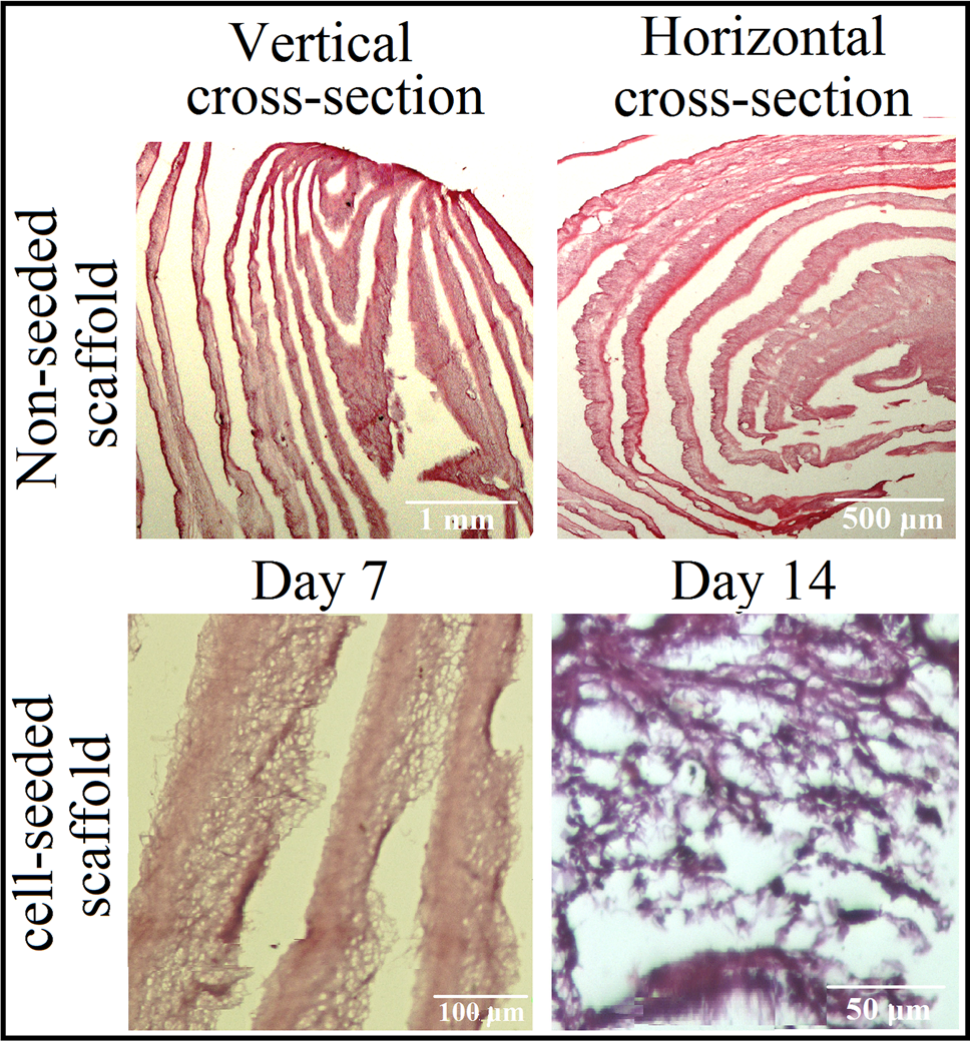
H&E staining images of vertical and horizontal sectioning of Roll E primary structures (*upper lane*) and cell seeded structures after 7 and 14 days (*second lane*)

To gain more understanding about micro structural properties of the prepared coral-loaded roll scaffold and diffusion state of the seeded cells histological study was done. After 7 and 14 days of In Vitro cell culture, coral-loaded scaffold (Roll E) was detached and fixed in formalin solution and vertical and horizontal sections from the midpoint between the top and bottom levels of cell seeded scaffold were prepared. Histological analysis with H&E staining, in addition to the illustration of distant layer by layer structure of the scaffold and sparse (non-compact) placement of nanofibers in each layer, showed MG-63 cells immigration within the scaffolds (Fig. 8).

### Matrix calcification

**Fig. 9:**
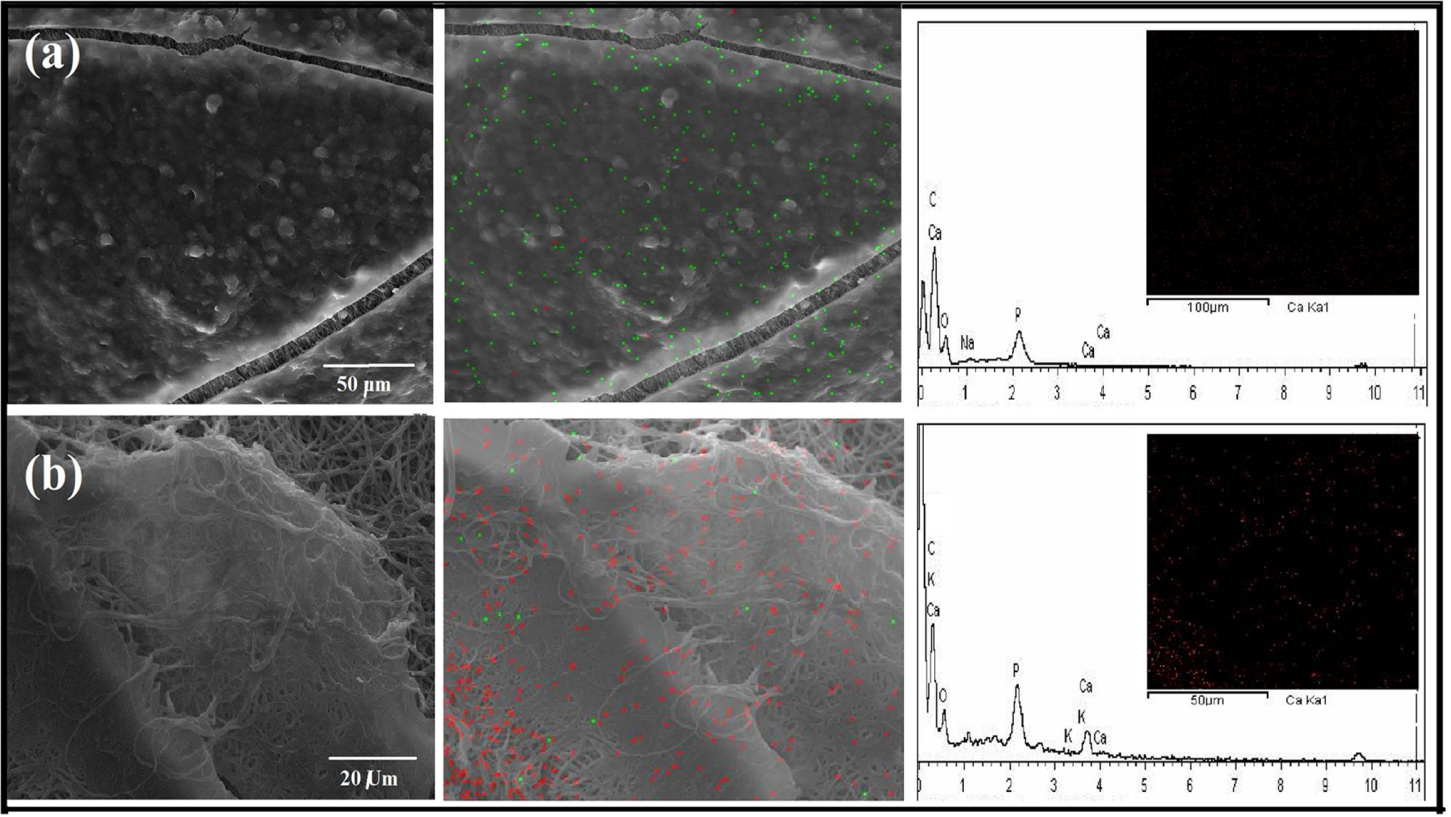
SEM images of seeded CF-Roll and Roll E scaffolds (**a**) and (**b**) after 14 days; and their respective distribution map of Ca (*red spots*) and P (*green spots*) over the scanned area, Together with their relative EDX elemental profile (color figure online)

Enhanced calcium deposition in the coral-loaded roll scaffold (Roll E) after 14 days was detectable in the EDXA distribution map of calcium (red spots) over the scanned area together with the related EDXA elemental profile [Fig. 8 (upper lane)], compared to that of coral-free structure [Fig. 9 (second lane)]. It is worth to mention that to prevent the effect of coral composition on the EDXA profile and Ca map, prior to this analysis analysis, coral microparticles were removed carfully from the polymeric substrate. Considering the Ca distribution map and Ca peak in EDXA elemental profile, the enhanced tendency of the cells to calcification is remarkable when they were seeded on coral-loaded roll scaffold.

## Conclusion

In this work, scaffolds for bone tissue regeneration were prepared by the use of electrospun PCL/Gelatin mats and natural coral microparticles. Existence of coral microparticles within the nanofibrous structure led to the enhancement in physical, mechanical and cellular characteristics. Significant increase in open and total porosity together with enhanced pore size of the coral-loaded rolls resulted in high water uptake value which indicates the possibility of nutrients and body fluid exchange of the related scaffolds with their surrounding environment. In addition, the resultant enhancement of the mentioned morphological and physical properties led to the good cellular infiltration, proliferation and mineralization within the scaffold. In vitro cytotoxicity evaluation revealed that no release of cytotoxic material from the scaffolds and coral particles occurred. Moreover, the fabricated structure showed comparable mechanical properties to that of natural cortical bone. Due to the reported results, the introduced roll-designed nanofibrous structure containing 200 wt% coral microparticles (with 200 μm average particle size) can be considered as an appropriate regenerative scaffold for load bearing bone defects.
